# Control of a wrist joint motion simulator: A phantom study

**DOI:** 10.1016/j.jbiomech.2016.07.001

**Published:** 2016-09-06

**Authors:** Darshan S. Shah, Angela E. Kedgley

**Affiliations:** Department of Bioengineering, Imperial College London, London, United Kingdom

**Keywords:** Wrist, Simulator, Kinematics, Control strategy, Muscle forces

## Abstract

The presence of muscle redundancy and co-activation of agonist–antagonist pairs in vivo makes the optimization of the load distribution between muscles in physiologic joint simulators vital. This optimization is usually achieved by employing different control strategies based on position and/or force feedback. A muscle activated physiologic wrist simulator was developed to test and iteratively refine such control strategies on a functional replica of a human arm. Motions of the wrist were recreated by applying tensile loads using electromechanical actuators. Load cells were used to monitor the force applied by each muscle and an optical motion capture system was used to track joint angles of the wrist in real-time. Four control strategies were evaluated based on their kinematic error, repeatability and ability to vary co-contraction. With kinematic errors of less than 1.5°, the ability to vary co-contraction, and without the need for predefined antagonistic forces or muscle force ratios, novel control strategies – hybrid control and cascade control – were preferred over standard control strategies – position control and force control. Muscle forces obtained from hybrid and cascade control corresponded well with in vivo EMG data and muscle force data from other wrist simulators in the literature. The decoupling of the wrist axes combined with the robustness of the control strategies resulted in complex motions, like dart thrower׳s motion and circumduction, being accurate and repeatable. Thus, two novel strategies with repeatable kinematics and physiologically relevant muscle forces are introduced for the control of joint simulators.

## 1 Introduction

Muscle activated physiologic simulators recreate the kinematic and kinetic conditions of a natural joint in cadaveric specimens by applying loads to the tendons. As in other joints of the body, redundant muscle activation occurs at the wrist because six primary muscles control its two degrees of rotation. Resolution of the load distribution between the muscles is vital to solve this indeterminate problem.

A common strategy for recreating joint motion has been to control one muscle, the ‘prime mover’, using prescribed excursion, while other muscles, classified as either synergists or antagonists, are simulated using prescribed forces. These forces are calculated as a proportion of the prime mover force, using some combination of physiological cross-sectional area (PCSA), lever arms, electromyographic (EMG) signals, and clinical knowledge of the muscles. This strategy has been used in shoulder (Kedgley et al., 2007), elbow (Johnson et al., 2000), forearm (Nishiwaki et al., 2014) and ankle (Sharkey and Hamel, 1998) simulators.

The most widely published wrist simulator employs a control strategy based primarily on position feedback; the agonists are controlled using a signal proportional to the error in joint position, whereas antagonists maintain a constant force (Werner et al., 1996). An alternative strategy has been to employ force control, where each muscle is controlled by a predefined set of force profiles corresponding to a specified motion (Erhart et al., 2012).

The aforementioned joint simulators employ assigned force profiles, or established muscle force ratios based on EMG and/or PCSA or muscle moment arms. Hence, they have predefined (Erhart et al., 2012) or unique (Werner et al., 1996) muscle force profiles for a given joint motion. However, redundant muscle actuation allows for the possibility of multiple force distributions resulting in the same kinematics, and for the occurrence of co-contraction – the ability of groups of muscles to produce higher forces simultaneously, in order to stabilize the joint. A computational study which combined both position and force control by an optimization technique to map joint torques to muscle forces suggests an additional method of joint simulator control, known as cascade control (Colbaugh and Glass, 1993). However, it has never been tried on a physiologic simulator.

The aims of this study were, therefore, to develop a repeatable muscle activated physiologic wrist simulator and to compare pre-established and novel control strategies. The hypothesis was that combining position and force feedback into one control algorithm would result in a more physiologic outcome.

## 2 Materials and methods

### 2.1 Design

**Fig. 1:**
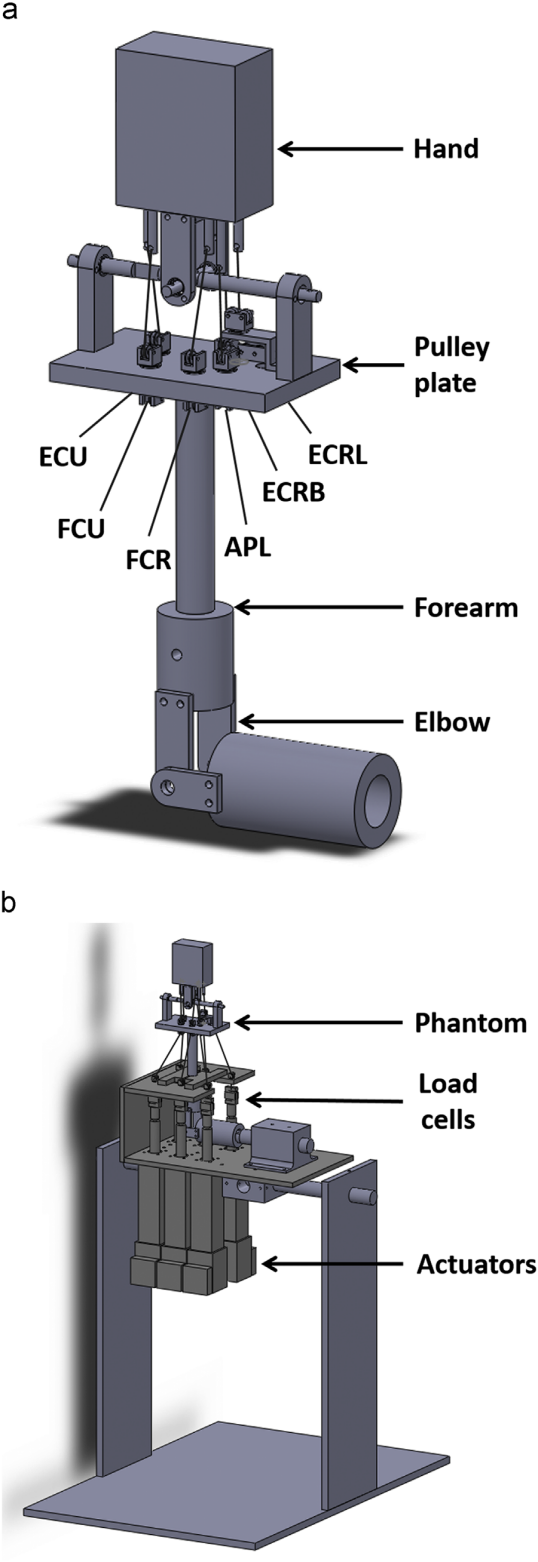
A schematic diagram of (a) the phantom and (b) the wrist simulator shown in the vertically upward position (FCR = flexor carpi radialis, FCU = flexor carpi ulnaris, ECRL = extensor carpi radialis longus, ECRB = extensor carpi radialis brevis, ECU = extensor carpi ulnaris, APL = abductor pollicis longus).

Motion at the wrist was recreated by applying tensile loads using linear actuators (SMS Machine Automation, UK) mounted in-line with servo motors (Animatics Corp., CA, USA) via steel cables guided through the custom pulleys (Fig. 1). Muscles with the greatest effect on the wrist (Brand and Hollister, 1999) were considered for this study – flexor carpi radialis (FCR), flexor carpi ulnaris (FCU), extensor carpi radialis longus (ECRL), extensor carpi radialis brevis (ECRB), extensor carpi ulnaris (ECU) and abductor pollicis longus (APL). Load cells (Applied Measurements Ltd., UK) were connected in series with the actuators to monitor force applied to each tendon. A six-camera optical motion capture system (Qualisys, Sweden) was used to obtain the joint angles in real time by placing clusters of reflective markers on the hand and forearm and following ISB recommendations for joint angle calculations (Wu et al., 2005). The wrist was driven using custom-written LabVIEW code (National Instruments, TX, USA) that implemented the algorithms discussed below. These algorithms were compared in a controlled manner on a phantom limb – an artificial, functional replica of a human hand and forearm (Fig. 1a).

The phantom hand replicated a 50th-percentile male hand with an open fist. The mass and center of gravity of the hand were calculated from anthropometric data (Tilley A.R. and Henry Dreyfuss Associates, 2002, Winter, 2009). Tendon insertions for each wrist tendon were obtained by digitizing their locations on a 3D model of the hand created by segmenting a CT scan of a model of the upper limb (Sawbones, WA, USA) using MIMICS 16.0 (Materialise, Belgium). Co-ordinates of all tendon insertions were calculated with respect to the head of the capitate, which was assumed to be the origin (Youm et al., 1978). A pulley plate, consisting of custom-made rotating pulleys on either side, served as the flexor and extensor retinacula. The pulleys were positioned to mimic the locations of the wrist tendons with respect to the flexion-extension (FE) and radioulnar deviation (RUD) axes in the transverse plane (Brand and Hollister, 1999). Decoupling the FE and RUD axes in the wrist facilitated the replication of complex functional motions, like dart thrower׳s motion (DTM) or circumduction.

### 2.2 Control strategies

Four different control algorithms were tested. Position and force control were established strategies used on previous wrist simulators, whereas hybrid and cascade control were novel strategies.

#### 2.2.1 Position control

**Fig. 2:**
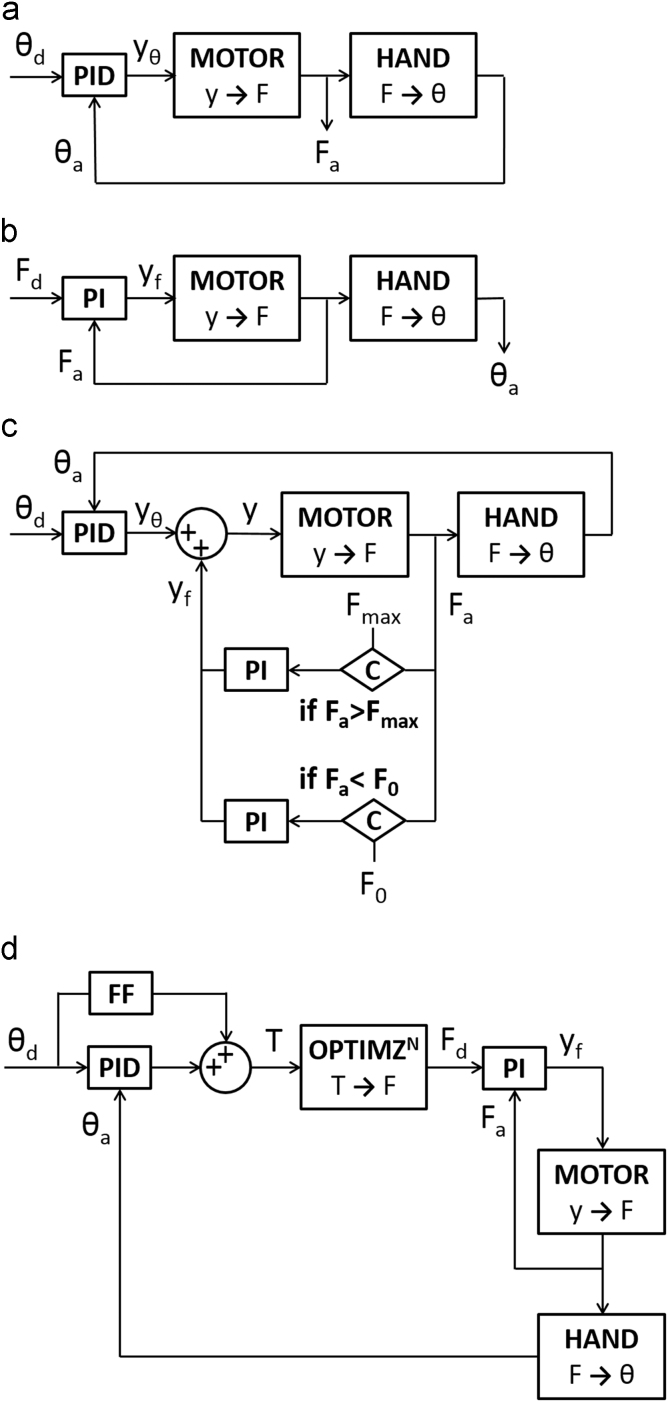
Block diagrams of the control strategies used on the wrist simulator (a) position control (b) force control (c) hybrid control (d) cascade control (*θ_a_*=actual joint angle, *θ_d_*=desired joint angle, *C*=comparator, *F_a_*=actual force, *F_d_*=desired force, *F_0_*=minimum force, *F_max_*=maximum force, PI=proportional-integral controller, PID = proportional-integral-derivative controller, *T*=joint torque, *y*=total actuator displacement, *y_θ_*=actuator displacement from position control, *y_f_*=actuator displacement from force control).

Errors between the desired trajectory and actual joint angles in FE and RUD were minimized using a proportional-integral-differential (PID) controller (Fig. 2a). Optimum PID parameters were obtained by carrying out Ziegler–Nichols tests (Ziegler and Nichols, 1995) and then manually adjusting them for low steady state error, high response time and low overshoot for a step input. From the kinematic error, the excursion of the ECRB was modified, while the excursions of the remaining tendons were driven by ratios of the moment arms (additional details in Appendix A.1). Muscle moment arms were determined by performing tendon excursion tests (An et al., 1983, Bremer et al., 2006, Kuxhaus et al., 2009) and were given as input for precise distribution of relative tendon excursion.

#### 2.2.2 Force control

Each actuator was given a custom force trajectory as the input, which reflected the force profile of the corresponding muscle for a specified motion, as determined from trials in position control. Error between the input force trajectory and actual cable forces from the load cells was minimized using a proportional-integral (PI) controller (Fig. 2b). The derivative term was kept at zero to stabilize the system (Datta et al., 2000).

#### 2.2.3 Hybrid control

Hybrid control was a combination of independent position and force control loops (Fig. 2c). The wrist was primarily moved under position control; however, if a muscle force fell outside a pre-set range, force control was applied to that muscle to ensure compliance with the minimum and maximum bounds. Defining minimum muscle forces (*F*_0_) prevented complete cable unloading and were specified based on the desired amount of co-contraction. Maxima (*F_max_*) were assigned by taking the product of the specific tension and muscle PCSA (Holzbaur et al., 2007). The specific tension was assumed to be constant at 25 N/cm^2^ (Kent-Braun and Ng, 1999, Narici et al., 1992).

#### 2.2.4 Cascade control

This control strategy, adapted from work by Colbaugh and Glass (1993), combined position control, force optimization, and force control (Fig. 2d). Two PID controllers computed the two torques – one each for FE and RUD – necessary to minimize the error between the desired and actual joint angles. A feedforward component was added to anticipate points of maximum acceleration in the motion. The following quadratic optimization routine, similar to those used in computational musculoskeletal models (Crowninshield and Brand, 1981), provided individual muscle forces necessary to obtain the joint torques, while the mechanical impedance controlled the amount of co-contraction.

$$\mathrm{OBJECTIVE: minimise}\sum_{i=1}^{6}{\left(\frac{F_{i}}{A_{i}}\right)}^{2}$$

$$\mathrm{such that}\sum_{i=1}^{6}r_{\mathrm{ij}}F_{i}=T_{j}$$

$$\sum_{i=1}^{6}F_{i}=\rho$$

$$F_{i}\geq 0$$

(where *i* = a muscle, *j* = FE or RUD, *F*_*i*_ = force in cable *i*, *A*_*i*_ = PCSA of muscle *i*, *r*_*ij*_ = moment arm of tendon *i* about axis *j*, *T*_*j*_ = torque about axis *j*, *ρ* = muscle impedance)

A set of six PI controllers, one for each muscle, minimized the error between the input force trajectory and actual cable forces.

### 2.3 Simulations

The phantom was placed with the wrist and forearm in the neutral position and elbow flexed to 90°. The simulator was used to move the hand in both vertically upward (hand above the elbow) and downward (hand below the elbow) positions using the aforementioned control strategies. The control strategies were evaluated based on low kinematic error, repeatability, and ability to vary co-contraction. The mean error in joint angles for planar motions – FE of amplitude 30° (FE-30) and RUD of amplitude 10° (RUD-10) – was used to compare the kinematics across control strategies. Co-contraction was controlled using *F*_0_ in hybrid control and *ρ* in cascade control. These parameters were held constant for the kinematic tests (*F*_0_=10 N, *ρ*=140 N) and varied for co-contractions tests. Non-planar cyclic motions – DTM (30° extension with 10° radial deviation to 30° flexion with 10° ulnar deviation) and circumduction (FE of amplitude 20° combined with RUD of amplitude 10°, in clockwise – flexion to ulnar deviation to extension to radial deviation – and anticlockwise – flexion to radial deviation to extension to ulnar deviation – rotations) – were also simulated using the acceptable control strategies. Each test was performed five times and the standard deviation of the mean kinematic error was used to quantify repeatability.

## 3 Results

**Fig. 3:**
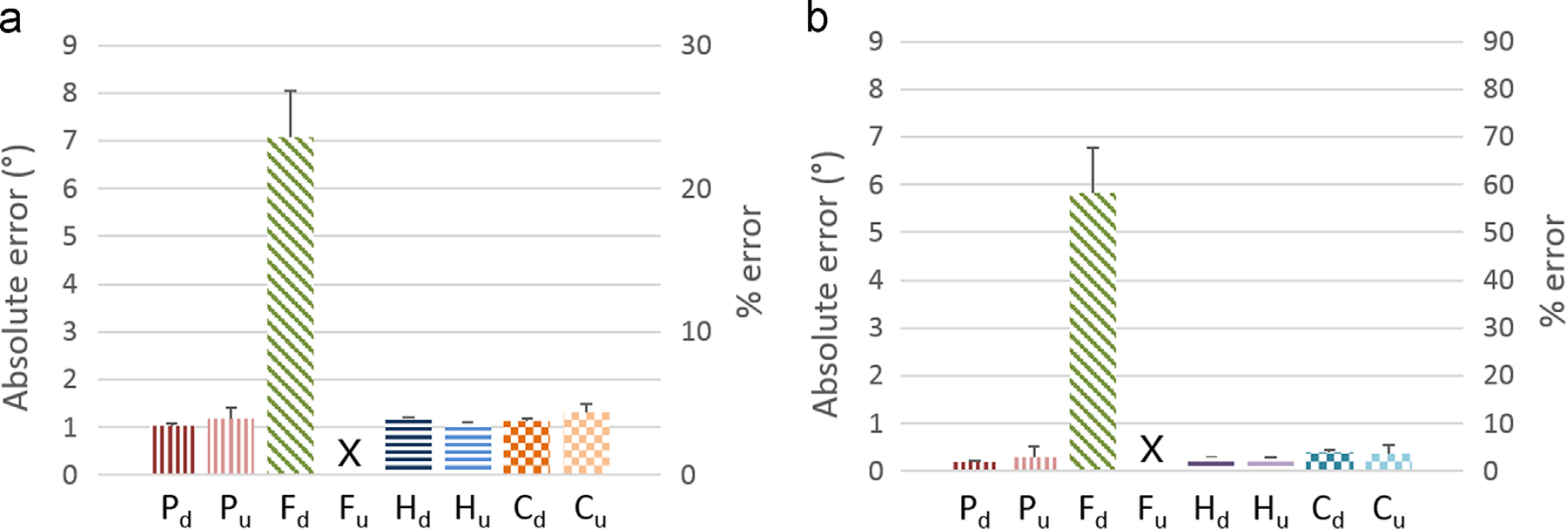
Mean in-plane kinematic error, reported as an absolute value and as a percent of the maximum range, across different control strategies (*P*=position control, *F*=force control, *H*=hybrid control, *C*=cascade control) for the hand in a vertically upward (u) and downward (d) position for two motions (a) FE-30 and (b) RUD-10. Force controlled motions in the vertically upward position, marked with an ‘X’, could not be completed.

Using force control, in the vertically downward position, mean errors of 7.1° and 5.8° resulted in the plane of motion during FE-30 and RUD-10 respectively, whereas in the vertically upward position, motions could not be completed (Fig. 3). Mean errors in the plane of motion for all other simulations were less than 1.5°. The low standard deviations associated with these control strategies illustrated the repeatability of the motions regardless of phantom orientation. Iteration time for cascade control was 10–12 milliseconds, as opposed to 4–5 milliseconds for the other control strategies.

**Fig. 4:**
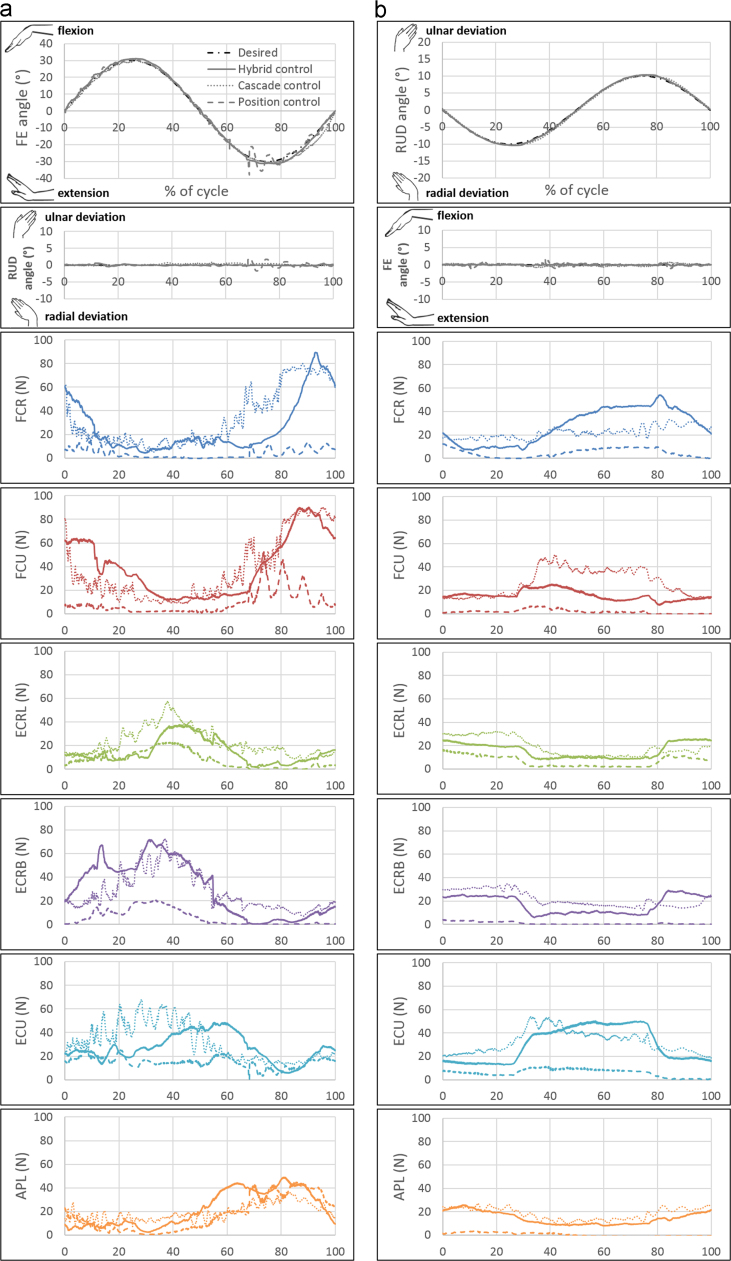
Kinematics in and out of the plane of motion as compared to desired kinematics (dot-dash lines) and typical muscle force profiles for position control (dashed lines), hybrid control (solid lines) and cascade control (dotted lines) with the hand in a vertically upward position for two motions (a) FE-30 and (b) RUD-10 (Flexion is positive, extension is negative, ulnar deviation is positive, radial deviation is negative according to ISB recommendations, FCR = flexor carpi radialis, FCU = flexor carpi ulnaris, ECRL = extensor carpi radialis longus, ECRB = extensor carpi radialis brevis, ECU = extensor carpi ulnaris, APL = abductor pollicis longus).

**Fig. 5:**
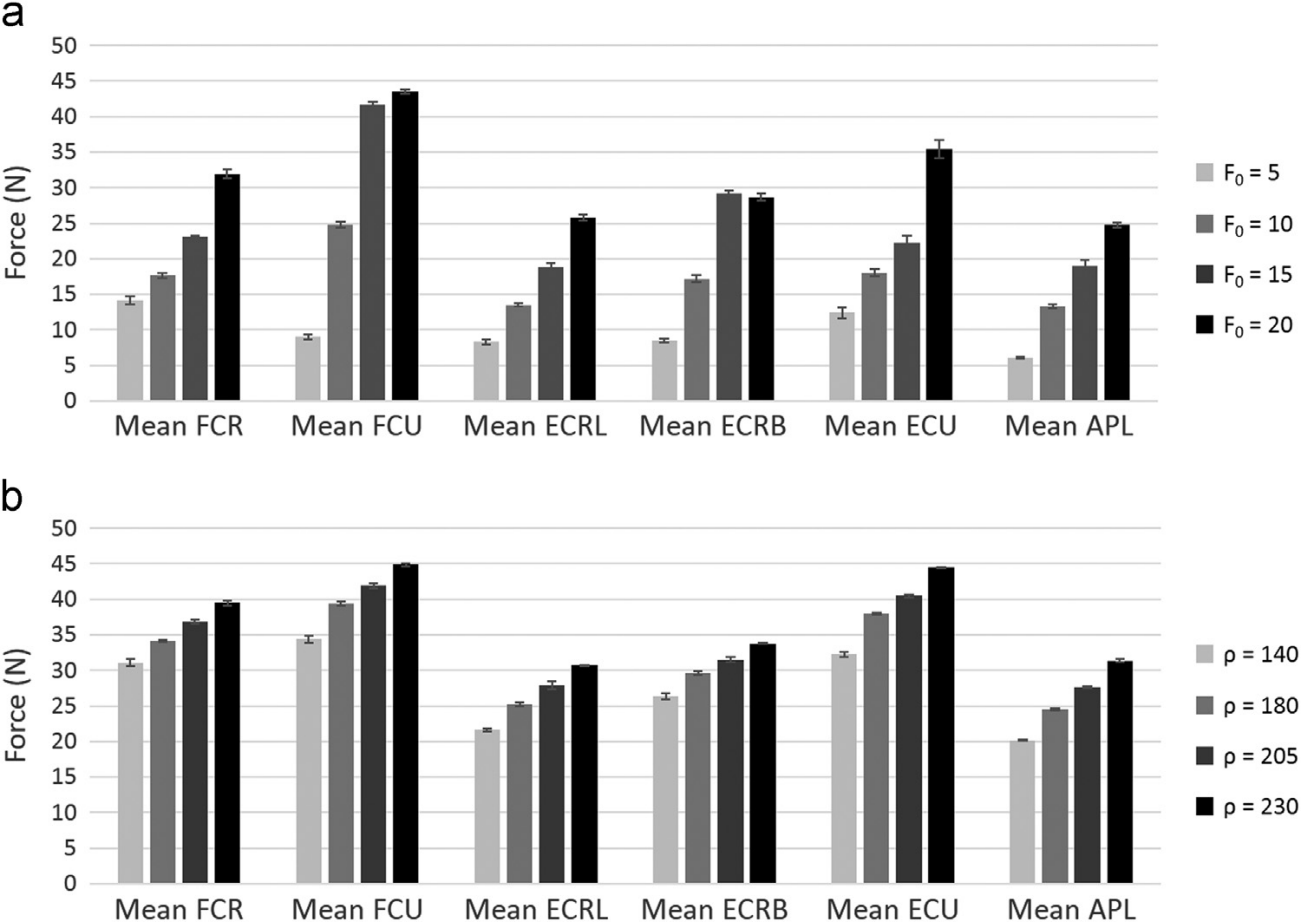
Comparison of mean muscle forces across varying co-contraction limits for FE-30 in (a) hybrid control (*F*_0_=lower bound on muscle forces) and (b) cascade control (*ρ*=muscle impedance) (FCR=flexor carpi radialis, FCU=flexor carpi ulnaris, ECRL=extensor carpi radialis longus, ECRB=extensor carpi radialis brevis, ECU=extensor carpi ulnaris, APL=abductor pollicis longus).

**Fig. 6:**
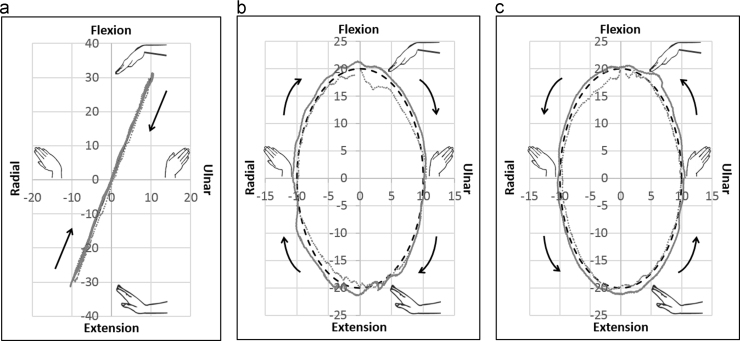
Trajectories of non-planar motions in hybrid (solid lines) and cascade control (dotted lines) as compared to the desired trajectory (dashed lines) (a) Dart thrower’s motion – 30° extension with 10° radial deviation to 30° flexion with 10° ulnar deviation. (b) Clockwise circumduction – 20° flexion to 10° ulnar deviation to 20° extension to 10° radial deviation. (c) Anticlockwise circumduction – 20° flexion to 10° radial deviation to 20° extension to 10° ulnar deviation (*X*-axis represents radioulnar deviation, *Y*-axis represents flexion-extension).

For FE-30, force trajectories for all the muscles in hybrid control were similar to those in cascade control (Fig. 4). As *F*_0_ and *ρ* were increased, in hybrid and cascade control respectively, both individual and mean muscle forces increased simultaneously over the entire range of motion (Fig. 5). Low kinematic errors both in and out of the plane for planar motion, as well as low kinematic errors for non-planar motions (Fig. 6) were observed using hybrid and cascade control (Table 1).

## 4 Discussion

A simulator was developed to replicate physiologic motions of the human wrist on a phantom limb. This phantom was designed to facilitate only two degrees of freedom – FE and RUD – although a small amount of pronation-supination is known to be present in the carpals (Foumani et al., 2009, Kobayashi et al., 1997). The six muscles, which insert at the base of the metacarpals – FCR, FCU, ECRL, ECRB, ECU and APL – were considered for this study. Extrinsic flexors and extensors of the fingers and the thumb were not included, despite the fact that they cross the wrist, primarily because they act on the finger joints, and hence, have smaller moment arms at the wrist. Moreover, since they pass through multiple joints before insertion, their inclusion would have added to the complexity of the control strategies. Steel cables were rigidly attached to the points of tendon insertions on the phantom (Appendix A.2).

Based on position feedback from an optical motion capture system and force feedback from load cells, four closed loop control strategies were implemented and tested. Mean kinematic errors and standard deviations for position, hybrid, and cascade control were less than 1.5° for both vertically upward and downward positions indicating good accuracy and repeatability (Fig. 3). The absolute errors were higher in FE-30 than RUD-10, owing to the larger range of motion; but when normalized with respect to the maximum amplitude of motion, percentage errors for position, hybrid, and cascade control were less than 5% for both motions. Force control, however, suffered from high kinematic errors in the downward position and was unable to perform the motions in the upward position. This was consistent with the results reported by Erhart et al. (2012). Thus, force control was eliminated as a possible control strategy. Although position control resulted in accurate motions with relatively lower forces, the hand was unstable in extension, since some muscle tendons were completely unloaded (Fig. 4). Hence, it was important to control muscle forces in order to prevent muscle tendons from unloading.

Hybrid control combined position and force feedback by applying bounds to muscle forces. Lower bounds were in agreement with EMG data, which shows that muscle activity in the neutral wrist position is almost 10% of the maximum voluntary contraction for forearm muscles (Fagarasanu et al., 2004). Specific tension in upper bounds, although held constant, has been shown to vary with physiological factors (Buchanan, 1995) and computational techniques (Maganaris et al., 2001).

Cascade control was adapted from a theoretical study and implemented on a joint motion simulator for the first time. The indeterminate problem of deciding muscle forces from joint torques was solved by employing real-time quadratic optimization. However, owing to this optimization block and the nested force control loop, cascade control had a longer iteration time, which in turn resulted in a higher cycle time as compared to hybrid control (Appendix A.1). The kinematic error in cascade control, although small (Fig. 3), was higher than that reported by Colbaugh and Glass (1993), since theirs was a computational study that did not simulate the practical variations between desired and actual forces (Fig. 2d). Muscle forces conformed well to published EMG data – in FE-30, ECRB produced a higher force than ECRL during extension (Tournay and Paillard, 1953), and all muscles worked synchronously, without any one of them acting as a prime mover (Backdahl and Carlsoo, 1961). However, the muscle force profiles were more oscillatory than those obtained for hybrid control, since optimum parameters in the various control blocks had to be selected to minimize both oscillations in muscle force profiles as well as kinematic error (Appendix A.3).

The level of co-contraction in the muscles of a joint directly affects the joint reaction force. EMG data indicates co-contraction and active antagonist forces for the wrist in various deviated positions (Fagarasanu et al., 2004). Hence, *F*_0_ and *ρ* were introduced as handles to vary co-contraction in hybrid and cascade control, respectively. By changing these handles, different force profiles for the same kinematic inputs were simulated. Co-contraction values for a joint depend on the external joint loading, and hence, vary considerably even for daily activities (Ricci et al., 2015). Therefore, instead of a single constant value of co-contraction, the motions for different co-contraction values were analyzed. However, one of the limitations of both hybrid and cascade control was that the level of co-contraction was defined in the control strategy using parametric handles i.e. the control strategies could not predict the level of co-contraction without additional parametrisation.

Determining antagonistic forces has been a limitation of many simulators reported in the literature. Antagonists either have pre-assigned force profiles (Erhart et al., 2012) or pre-established muscle ratios (Johnson et al., 2000, Kedgley et al., 2007, Sharkey and Hamel, 1998) or are held constant over the entire range of motion (Werner et al., 1996). Moreover, for complex motions like DTM and circumduction, it is very difficult to identify the antagonists. The control algorithms in hybrid and cascade control eliminated the need to differentiate muscles as agonists and antagonists during a motion. Variable antagonistic forces thus obtained were more physiologically realistic. Peak forces of all muscles conformed well to those reported in previous studies (Dimitris et al., 2015, Erhart et al., 2012, Farr et al., 2013, Werner et al., 1996, Werner et al., 2010).

In conclusion, a wrist simulator was designed to replicate planar as well as complex wrist motions accurately and repeatably. Novel control strategies – hybrid and cascade control – were used to achieve variable co-contraction in the wrist for the first time and were preferred over other control strategies. In future, the phantom hand will be replaced with cadaveric specimens for study of surgical procedures on the wrist.

## Conflict of interest statement

The authors have no conflicts of interest to declare.

## Figures and Tables

**Table 1:** Mean±standard deviation of kinematic error and maximum joint angles during a selection of cyclic motions in hybrid and cascade control (FE-30 = flexion extension of ±30°, RUD-10 = radioulnar deviation of ±10°, DTM = dart thrower’s motion from 30° extension with 10° radial deviation to 30° flexion with 10° ulnar deviation, CCDcw = clockwise circumduction from 20° flexion to 10° ulnar deviation to 20° extension to 10° radial deviation, CCDacw = anticlockwise circumduction from 20° flexion to 10° radial deviation to 20° extension to 10° ulnar deviation (standard deviation of less than 0.05 has been reported as 0.0).

Motion	Control strategy	Mean error in FE (°)	Mean error in RUD (°)	Max. flexion (°)	Max. extension (°)	Max. ulnar deviation (°)	Max. radial deviation (°)
FE-30	Hybrid	1.1±0.0	0.1±0.0	31.5±0.1	31.8±0.3	0.7±0.0	0.5±0.0
	Cascade	1.2±0.2	0.3±0.0	30.6±0.1	30.9±0.3	0.9±0.1	0.5±0.2
							
RUD-10	Hybrid	0.1±0.0	0.3±0.0	0.6±0.1	0.5±0.1	10.4±0.0	10.5±0.0
	Cascade	0.2±0.0	0.4±0.0	0.9±0.1	0.9±0.1	10.1±0.1	10.2±0.0
							
DTM	Hybrid	1.0±0.3	0.3±0.1	31.8±0.3	31.5±0.2	10.7±0.1	10.5±0.0
	Cascade	0.8±0.1	0.6±0.0	29.9±0.2	29.6±0.3	9.9±0.3	9.4±0.1
							
CCDcw	Hybrid	0.9±0.0	0.3±0.0	21.6±0.2	21.8±0.1	10.5±0.1	10.5±0.0
	Cascade	0.7±0.1	0.8±0.2	19.7±0.1	20.2±0.3	10.9±0.6	10.2±0.1
							
CCDacw	Hybrid	0.8±0.0	0.4±0.0	21.6±0.2	21.5±0.1	10.6±0.0	10.5±0.0
	Cascade	0.6±0.0	0.6±0.0	20.0±0.2	20.3±0.1	9.9±0.1	9.8±0.1
