# Supplementary material for: Control of a wrist joint motion simulator: A phantom study
Source: J Biomech. 2016 Sep 6;49(13):3061–8. doi: 10.1016/j.jbiomech.2016.07.001 (PMC5061070; doi:10.1016/j.jbiomech.2016.07.001)
Supplement: Supplementary file 1 — Supplementary material [file mmc1.pdf]

Control of a wrist joint motion simulator: a phantom study

Darshan S Shah, Angela E Kedgley

Appendix A: Supplementary information

A.1 Additional points regarding the methodology

In position control, the excursion of the extensor carpi radialis brevis (ECRB) was modified based on the kinematic error, while the excursions of the remaining tendons were driven by ratios of the moment arms. The ECRB was chosen to be the 'master' tendon since it has the largest moment arm in flexion-extension amongst all the extensors. Moreover, since it passes through a dedicated compartment in the extensor retinaculum, the ECRB has a constant moment arm throughout the range of motion (Brand and Hollister, 1999). However, no particular tendon was chosen to be a prime mover, i.e. that which has the greatest effect in producing a specified motion; the method of assigning a 'master' tendon was employed only to reduce the mathematical calculations in the control algorithm.

The sample rate for the optical motion tracking system (Qualisys, Sweden) was 50Hz. The sample rate was 160Hz for the load cells (Applied Measurements Ltd., UK). The cycle times for various control strategies are listed in Table A1. Force control resulted in a high kinematic error in flexion-extension and radioulnar deviation, despite high cycle times.

Control strategy	Cycle time (sec)
Position control	10
Force control	60
Hybrid control	10
Cascade control	30

Table A1: Period of cycle of motions in various control strategies

A.2 Compliance in tendons

Tendons of the wrist muscles have a much lower Young's modulus of elasticity (E≈500MPa) (Loren and Lieber, 1995) as compared to that of steel (E=200GPa). One of the limitations of our phantom, therefore, was the rigid attachments of the steel cables to the hand at the points of tendon insertion, as opposed to the more compliant tendinous attachments in the human hand. To observe the effects of this additional compliance on our control strategy, an HDPE spacer (E=404MPa) was connected in series with the steel cable while tuning the cable in force control for a step input of 30N. Although such a high step would never be experienced by an actuator during a single iteration of the simulation of cyclic motions of the wrist, it was chosen to amplify the effect of compliance as well as check the robustness of the controller. The optimum proportional-integral (PI) parameters were obtained using the Ziegler-Nichols test (Ziegler and Nichols, 1995), while the derivative term was kept at zero to stabilize the system (Datta, Ho and Bhattacharya, 2000). As compared to the optimum PI parameters obtained for a rigid steel cable attachment, the compliant attachment with the HDPE spacer resulted in an increase in the proportional gain by 17%, while the integral gain remained constant. Although the steady state error reduced from 1.2N in the rigid attachment to 0.7N in the compliant attachment, both values were within acceptable limits of 5% of the step input. Thus, improvements observed in optimum PI gains as well as steady state error for the compliant attachment were not significant. Moreover, one of the drawbacks of using HDPE spacers was the reduction in available excursion of each tendon, since they were attached in series with the steel cable. Therefore, we decided to connect steel cables directly to the points of tendon insertion.

A.3 Force profiles in cascade control

Muscle force profiles in cascade control were more oscillatory as opposed to the smoother profiles in hybrid control owing to a higher proportional gain in the force control 'PI' block of cascade control (Fig. 2d). If this gain was decreased, in an attempt to reduce the observed turbulence, the actuators took longer to reach their allotted force. Since the force 'PI' loop was nested inside the position 'PID' loop (Fig. 2d), this resulted in an increase in the time required to correct the joint angles, which resulted in an increase in the kinematic error. Alternatively, if the proportional gain in the position control 'PID' block was increased to correct the angular position error faster, this increased the required torques, which in turn increased the forces, resulting in an increase in the kinematic error. In summary, since cascade control employed force control nested in position control, optimum parameters in the various control blocks had to be selected to minimise both oscillations in muscle force profiles as well as kinematic error.

References

Brand P.W. and Hollister A.M., Mechanics of Individual Muscles at Individual Joints. In: *Clinical Mechanics of the Hand.* 3rd edition, 1999, Mosby, Inc.; St. Louis, pp.100-183.

Datta A., Ho M.T. and Bhattacharya S. P., *Advances in Industrial Control: Structure and Synthesis of PID Controllers*, 2000, Springer-Verlag; London.

Loren G. and Lieber R., Tendon biomechanical properties enhance human wrist muscle specialization, *J. Biomech.* 28 (7), 1995, 791-799.

Ziegler J. and Nichols N., Optimum settings for automatic controllers, *INTECH.* 42 (6), 1995, 94-100.
